# Identification of Heavy Tobacco Smoking Predictors-Influence of Marijuana Consuming Peers and Truancy among College Students

**DOI:** 10.3390/healthcare9121666

**Published:** 2021-12-01

**Authors:** Mihaela-Daiana Popa, Abhinav Sharma, Nilima Rajpal Kundnani, Otilia Lavinia Gag, Ciprian Ilie Rosca, Valeria Mocanu, Anca Tudor, Ramona Amina Popovici, Brigitha Vlaicu, Claudia Borza

**Affiliations:** 1 Department of Microbiology, University of Medicine and Pharmacy ‘Victor Babes’, 300041 Timisoara, Romania; popa.mihaela@umft.ro; 2 Department of Cardiology, University of Medicine and Pharmacy ‘Victor Babes’, 300041 Timisoara, Romania; sharma.abhinav@umft.ro; 3 Department of Occupational Medicine, Municipality Hospital, 310030 Arad, Romania; 4 Civil Medical Society Dr Rosca, 307405 Teremia Mare, Romania; roscaci@yahoo.com; 5 Department of Functional Sciences, Physiology, Centre of Immuno-Physiology and Biotechnologies (CIFBIOTEH), University of Medicine and Pharmacy ‘Victor Babes’, 300041 Timisoara, Romania; 6 Depatment of Dentistry, Faculty of Dental Medicine, ‘Vasile Goldis’ Western University of Arad, 310025 Arad, Romania; stana.otilia@gmail.com; 7 Discipline of Management, Legislation, and Communication in Dental Medicine, Faculty of Dental Medicine, University of Medicine and Pharmacy ‘Victor Babes’, 300041 Timisoara, Romania; ramona.popovici@umft.ro; 8 Department of Internal Medicine-Medical Semiotics, Centre for Advanced Research in Cardiovascular Pathology and Hemostasis, University of Medicine and Pharmacy ‘Victor Babes’, 300041 Timisoara, Romania; 9 Department of Ophthalmology, University of Medicine and Pharmacy ‘Victor Babes’, 300041 Timisoara, Romania; valeria_mocanu@yahoo.com; 10 Department of Functional Science-Biostatistics, University of Medicine and Pharmacy ‘Victor Babes’, 300041 Timisoara, Romania; anca.ancutza@gmail.com; 11 Department of Hygiene, University of Medicine and Pharmacy ‘Victor Babes’, 300041 Timisoara, Romania; vlaicu@umft.ro; 12 Department of Functional Science-Physiopathology, Center for Translational Research and Systems Medicine, University of Medicine and Pharmacy ‘Victor Babes’, 300041 Timisoara, Romania; claudia_borza@yahoo.com

**Keywords:** college students, smoking predictors, marijuana use, truancy, heavy smoker status, associated factors

## Abstract

Background: Poorly informed college students tend to adopt the habit of cigarette smoking. This habit often continues into their adulthoods, adversely affecting the population’s health and increasing the burden on healthcare systems. Aim: We aimed at exploring the predictors of the avoidable habit of smoking. We performed an analysis of the correlation between the potential predictors (marijuana use among peers and truancy) and the tobacco smoking statuses of the students. Material and method: Our study sample included 2976 students from colleges in Timis County, Romania, during the 2018–2019 period. The gender distribution of the participants was 62.5% girls and 37.5% boys, between the ages 18 and 25 years. A logistic regression test was performed to determine the impact of some personal and environmental factors, which are responsible for heavy smoking in this population. Results: Our findings suggest that the degree of marijuana smoking among friends and the frequency of college truancy are meaningful predictors of heavy smoking among young adults. The students with higher cigarette smoking rates had significantly more marijuana-smoking friends when compared to the students with average smoking rates. The truancy was higher among the students with higher cigarette smoking rates, compared to the students with average smoking rates.

## 1. Introduction 

Around the world tobacco consumption among young adults has reached epidemic levels, negatively impacting the health of populations and increasing financial burden on the healthcare systems [1,2,3]. The majority of young smokers tend to continue smoking later into their adulthoods. Half of adult smokers die prematurely from a smoking-associated pathology [4,5,6,7,8,9].

Though numerous programs have been implemented to curb smoking and thousands of articles have been written to warn against the use of tobacco, a substantial number of individuals across generations continue using this hazardous product. The use of tobacco usually begins during childhood and adolescence [10,11], with 88% of grown-ups reporting to have initiated smoking before the age of 18 years [12]. Owing to immaturity, college students are considered impressionable, increasing their likelihood of being influenced by the media showing advertisements on tobacco use, which often feature attractive models/movie actors portraying smoking as “cool”. Tobacco dependence remains a serious problem on a global scale. Among the individuals that once tried to smoke, approximately a third will become daily smokers [11]. Smokers trying to stop will succeed around 5% of the time. Even though not all smokers will become addicted to nicotine, the proportion of people diagnosed with nicotine addiction is higher than for any other substance [13,14]. Nicotine belongs to a large class of substances called alkaloids which contain an amine nucleus.

The chemical name of nicotine is (S)-3-(1-Methyl-2-pyrrolidinyl) pyridine, which reflects the existence of two cycles of carbon atoms that also contain nitrogen. In pure form, nicotine is a colorless or slightly yellowish oily fluid. Chemically, nicotine is a base that combines with acids to form water-soluble stable salts. Nicotine is absorbed fastest by cigarette smoke inhalation and the highest arterial blood values are reached within 20 s of inhalation. In body fluids, such as blood, most nicotine molecules are positively charged and that is why they cannot cross cell membranes by themselves. The positive charge is the result of nicotine’s chemical composition—a weak base with a pH of approximately 8.0 [15]. There is, however, a significant proportion of nicotine, about 30%, which travels without any electrical charge, and this form reaches the brain and other target tissues easily. Apart from nicotine, tobacco and cigarette smoke contain certain other ingredients such as Nornicotine and Acetylaldehyde. These ingredients either exert synergistic effects or potentiate the effect of nicotine. Certain active metabolites of nicotine have been noticed to exert strong effects on the central nervous system after acute nicotine administration [16]. 

Nornicotine is both a metabolite of nicotine and a minor alkaloid in tobacco. At low nornicotine concentrations, nicotine receptor antagonists, such as mecamylamine and [3H]-dihydro-β-eritroidine (DHβE) inhibit *S* (-)-nornicotine-triggered dopamine release. At high nornicotine doses, this inhibition is absent, indicating that high doses of dopamine release can be triggered by non-selective mechanisms. *S* (-)-nornicotine, *R* (+)-nornicotine, and nicotine are all involved in activating the neuronal mechanisms responsible for behavior sensitization [17].

Nicotine is extensively metabolized in the body, mainly in the liver. In humans, there are six primary metabolites [15]. Nicotine is the major component of tobacco and cigarette smoke (approximately 7–8 mg/cigarette). Nicotine can be measured, both in active and passive smokers, in several body fluids including serum, urine, and saliva. 

Cotinine, formed as a result of nicotine oxidation by P450 cytochrome, is one of nicotine’s primary metabolites. Having a blood half-life of fewer than two hours, the levels of nicotine concentration in biological fluids can help determine the recentness of exposure. By contrast, Cotinine has a blood or plasma half-life of 15–19 h. An international study, conducted across six countries by Biber et al. [18], compared the analytical results of urine and serum cotinine. The results of the study indicated that both gas chromatography (GC) and RIA (radioimmunoassay) accurately assess the quantities of cotinine in serum and blood samples.

In cancer, a common assessment of the biologically effective dose of nicotine is obtained by measuring the DNA adducts’ levels. Several mutagens and carcinogens are metabolically activated in vivo to electrophilic forms capable of interacting with cellular macromolecules. One of the mechanisms used by the body to fight the electrophilic attack is to conjugate reactive chemical residues with reduced glutathione, which is a nucleophile. This reaction induces an increase in polar thioether conjugates which are excreted from the body through urine and bile. Thioether urine concentrations are used as an unspecific indicator of exposure to alkylating agents. Smoking has been noticed to induce a dose-dependent increase in thioether urine excretion. Chemical substances found in cigarette smoke, excreted in urine as thioethers, include benzene, styrene, and vinyl chloride [19]. High concentrations of alkyl adenines and alkyl guanines from the reaction of alkylating agents with DNA have also been found in the urine of smokers. All three types of carcinogens (thioethers, alkyl adenines, alkyl guanines) reflect the ratio and balance between activation and detoxification [20].

The chemical structure and pharmacology of nicotine qualify it as the drug that creates the strongest addiction. Tobacco products are created to enable nicotine’s fast extraction, absorption, and distribution to the central nervous system. This drug is 5 to 10 times more potent than cocaine or morphine in generating physical and behavioral signs associated with addiction, including pleasure and preference [21]. The development of nicotine addiction depends on the quantity of nicotine that enters the body and its mode of delivery; rapid intake, absorption, and attainment of high plasma concentration increase the potential of creating addiction [22].

The understanding of the consequences of health statuses and pathologies induced by smoking has prompted the scientific foundations to increase awareness on prevention and cessation of both active and passive smoking. In this context, this study aimed at exploring the predictive behaviors/habits suggestive of the degree of tobacco use among young college students, and other elements in their immediate environment that may influence not only the smoker/nonsmoker status but also the smoking intensity. 

## 2. Materials and Methods 

Our sample consisted of 2976 college students from different universities within the Timiş County, residing in a typical Romanian urban environment in the academic year 2018–2019. The sample comprised 62.5% girls and 37.5% boys, between the ages of 18 and 25 years. The majority of students were 21 years of age (27.2%). We performed a cross-sectional population test, based on the CORT 2004 inventory which explores hazardous behaviors that impact the health of teenagers and young people alike, conducted during a type A CNCSIS survey [23].

CNCSIS (Consiliul National al Cercetarii Stiintifice din Invatamantul Superior), Romania, is a “national council for scientific research and superior studies” which conducts studies and surveys for research purposes. 

The CORT 2004 inventory is a 126-item questionnaire that was to be answered within 60 min. It entailed questions covering health risk behaviors such as nutrition habits, family environment, sexual behaviors, substance use, aggressiveness, physical activity, and depression. Participation in the survey was voluntary and anonymous.

The design of the CORT 2004 inventory was based on the following Romanian and international studies: (a) The American Study Monitoring the Future, (b) The European study ESPAD (The European School Project on Alcohol and Drugs), (c) The American study YRBSS (Youth Risk Behavior Surveillance System), and (d) The Timis County CAST study (Use of Alcohol, Drugs, and Tobacco).

Ethical clearance was obtained from the Ethical committee, Timisoara, Romania, prior to conducting the survey. Informed consent was obtained from the students prior to their inclusion in the study. 

### Statistical Analysis 

Statistical analysis was performed with SPSS software (version 17, SPSS Inc., Chicago, IL, USA). Comparisons between numerical series were performed with a nonparametric Mann–Whitney U test in case of comparisons between two series of values with non-Gaussian distribution and between more than two groups’ comparisons we applied a Kruskal–Wallis test. Logistic regression was used to identify all potential predictors for the heavy smoker status. For nominal variables, frequency tables were elaborated and the associations between these were achieved by applying the chi2 (χ^2^) test. We used the value of *p* < 0.05 for significant differences/associations.

## 3. Results 

### 3.1. Heavy Smoker Status

A logistic regression test was performed to determine the impact of some personal and environmental factors, on the heavy smoker status (smoking rate increased to more than 10 cigarettes/day) among college students. 

The assessment model consisted of 19 independent variables (gender, last school graduated by the father, last school graduated by the mother, satisfaction regarding the family’s financial situation, number of smoking friends, number of friends using alcohol, number of marijuana-smoking friends, the smoking statuses of parents and siblings, number of days the individual skipped school, current educational situation, age of first cigarette, the desire to stop smoking, number of days practicing binge-drinking, marijuana consumption, feelings of sadness, suicidal thoughts, knowledge about adverse effects of smoking). The model containing these predictors are statistically significant (χ^2^ Test, *p* < 0.001). Evaluation based on these variables demonstrated that the proposed model can help differentiate between smoking and nonsmoking students. The model used can explain the difference between 21.7% and 30.0% of variations in the heavy smoker status and can accurately classify 73.8% of cases. The most statistically significant predictors were the increased number of marijuana-smoking friends and the frequency with which students skipped college/university (Table 1).

### 3.2. Marijuana Consumption among Peers 

Around 10.8% of friends of nonsmoking students consumed marijuana, while 88.1% had no marijuana-smoking friends. Among the smoking students, 28.1% had marijuana consuming friends, while the remaining 70.8% among the smoking group had no marijuana-smoking friends. Hence, it can be stated that the smoking students had significantly more marijuana-smoking friends (28.1%), compared to the nonsmoking students (10.8%) − χ^2^.

The masculine gender represents a significant predictor for nonsmokers (the protective factor for the heavy smoker, OR = 0.590, 95% C.I. for OR is (0.350, 0.992)).

The status smoker of the mother (Yes) represents a significant predictor for nonsmokers (the protective factor for the heavy smoker, OR = 0.607, 95%C.I. for OR is (0.373, 0.988)).

The higher number of smoking friends represents a significant predictor for smokers (the risk factor for the heavy smoker, OR = 2.105, 95%C.I. for OR is (1.402, 3.159)).

Additionally, the increased number of marijuana-smoking friends represents a significant predictor for smokers (the risk factor for the heavy smoker, OR = 1.733, 95%C.I. for OR is (1.015, 2.960)).

The higher number of days since individual skipped school represents a significant predictor for smokers (the risk factor for the heavy smoker, OR = 1.468, 95%C.I. for OR is (1.228, 1.754)).

The presence of attempts at stopping smoking represents a significant predictor for smokers (the risk factor for the heavy smoker, OR = 1.763, 95%C.I. for OR is (1.033, 3.008)).

Additionally, the presence of suicidal thoughts represents a significant predictor for smokers (the risk factor for the heavy smoker, OR = 2.184, 95%C.I. for OR is (1.092, 4.370)).

In the smoking students’ category, we found that the smoking intensity is influenced by the number of marijuana-smoking friends (Mann–Whitney U test, *p* < 0.001). 

We found that students with increased smoking rates have significantly more marijuana-smoking friends, compared to students who smoke at an average rate (Mann–Whitney U test, *p* = 0.002) and that there’s no difference between students smoking at an average or low rate in terms of the number of marijuana-smoking friends.

### 3.3. Absenteeism over A Period of 30 Days—“Truancy Percentage”

In the nonsmoking student group, 42.5% did not skip a single day, 26.6% skipped one day, and 21.9% skipped 2 to 4 days. Around 4.3% of the nonsmoking students skipped over a week. In the smoking group, a huge percentage of 29.3%, skipped 2 to 4 days, 21.8% skipped a day and 11.3% skipped over a week.

**Figure 1:**
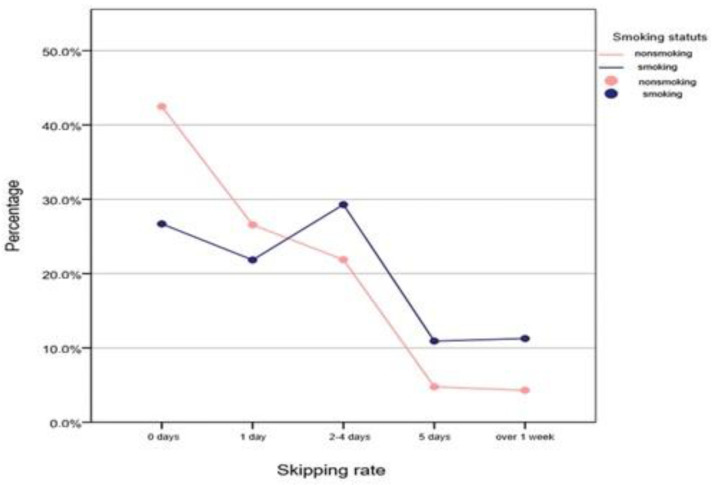
Percentage distribution of students in function of the number of days skipped and smoking.

We found that the smoking student group has skipped significantly more days than the nonsmoking student group (Mann–Whitney U test, *p* < 0.001—Figure 1).

In the smoking student group, we found that the smoking intensity was influenced by the number of days they have skipped (Mann–Whitney U test, *p* < 0.001). 

**Figure 2:**
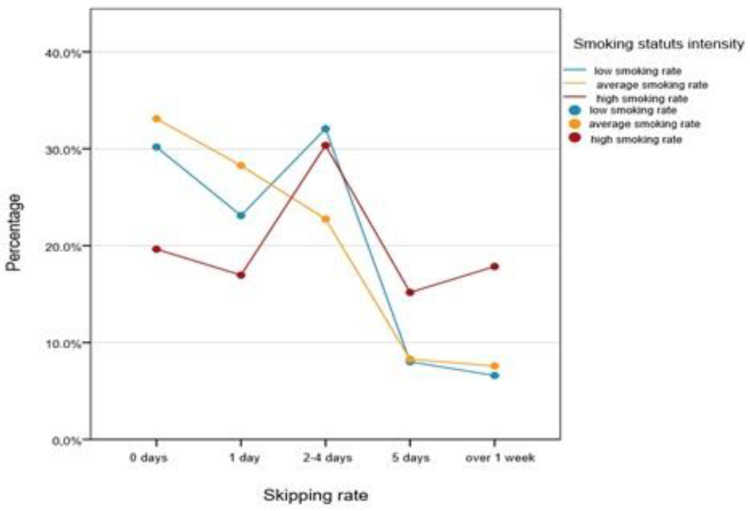
Percentage distribution of smoking students as a function of the numbers of days they skipped and the smoking intensity.

We compared the percentage of students in function of the numbers of days they skipped and the smoking intensity with the Kruskall–Wallis Test, and the differences were significant (*p* = 0.003). We found that the students with higher smoking rates have skipped school significantly more, compared to students with average smoking rates (Mann–Whitney U test, *p* < 0.001), and that there is no difference between students smoking at an average or low rate in terms of the numbers of skipped days (Figure 2). 

## 4. Discussion

A number of cross-sectional and longitudinal studies [24,25] have shown that the smoking habit of the peers and the perception of that habit is associated with the smoking behavior of the fellow student. Similarity or homogeneity of the tobacco consumption pattern among college students and their friends have led researchers [26,27] to conclude that the environment of the friends influences the smoking habits of the individuals. The most common mechanism reported is social learning [28], through which college students learn about the use of tobacco by observing their smoking friends. Young adults perceive smoking as a means to be socially accepted in a social environment and or cement an apparent social identity. Other mechanisms include peer pressure and the offer of cigarettes and other tobacco products [29].

Several models have been proposed to prevent the initiation of smoking in young adults. Models such as “The Information Deficit Model” that aim at providing information about the adverse health effects of smoking to young students [11,30]. Similarly, “The affective education model” can be used to help students develop social competence and stronger intrapersonal resources. This model is used to increase students’ level of self-esteem and improve their attitudes toward school, community, and family [11]. A third model, known as “The Social Influence Model” mainly focuses on the psychosocial factors related to smoking initiation, e.g., smoking in the immediate environment, peer smoking, and other social psychological factors [31]. 

Along with addiction, various sociodemographic factors tend to influence a person’s behavior and attitude towards tobacco-free and smoke-free policies. On-campus health promotion initiatives can minimize tobacco use among young adults. However, instituting policies or programs to reduce on-campus smoking is challenging in terms of fighting tobacco companies’ on-campus influence and protests from members of the college community against all indoor and outdoor smoking bans. Policy implementation often encounters a conflict between the agreeing and disagreeing students [32]. Successful implementation of preventive programs and policies entails the resolution of these conflicts to prioritize public interests and avoid social threats [33].

In addition to peer influence, the indicator of school absenteeism can also be used to screen potential student smokers, a finding that was also highlighted by a study conducted in Scotland, in which truant students were found to be twice more likely to become daily smokers [34]. 

## 5. Limitations of The Study 

The sample size in our study was substantial, and the results are noteworthy. However, due to the cross-sectional nature of the study, the associations reported in the study do not reflect causal relationships. Furthermore, due to the limited resources and the focus of our study, we explored just the unidirectionality of the marijuana and tobacco smoking relationship. 

## 6. Conclusions 

Our study concluded that marijuana consumption among peers and truancy are powerful predictors of heavy smoking among young students. The findings of our study indicate that smoker students have a significantly higher number of marijuana-smoking friends when compared with non-smoker students. Similarly, heavy-smoker students have a substantially higher number of marijuana smoking friends than average-smoker students. However, no correlation was found between the average or low smoker students and the number of marijuana-smoking friends.

Furthermore, significant truancy was noticed within the smoking student group versus the nonsmoking student group. Students with higher smoking rates skipped significantly more school days, compared to students with average smoking rates. However, no correlation between average or below average smoking students and truancy was found.

Identification of the tobacco smoking habit predictors can help us implement cessation programs and institute early interventions, like providing information about the adverse health effects of cigarette smoking, helping students increase their level of self-esteem, and improve their attitudes toward school, community, and family. Moreover, the use of models that focus on psychosocial factors related to smoking initiation, such as peer smoking and other social and psychological factors can help prevent the influx of new young smokers.

## Figures and Tables

**Table 1:** Logistic regression to determine the heavy smoker status of college students using smoking as a dependent variable.

Variables in the Equation	B	S.E.	Wald	df	Sig.	Exp(B)	95% C.I. for EXP(B)	
							Lower	Upper
Sex (M)	−0.528	0.266	3.95	1	0.047 *	0.590	0.350	0.992
Last graduated school of the father	−0.116	0.128	0.83	1	0.362	0.890	0.693	1.143
Last graduated school of the mother	−0.056	0.134	0.17	1	0.674	0.945	0.727	1.229
Satisfaction regarding the family’s financial situation	−0.117	0.123	0.90	1	0.342	0.890	0.699	1.133
Smoking status of the father (Yes)	−0.123	0.239	0.26	1	0.606	0.884	0.554	1.412
Smoking status of the mother (Yes)	−0.499	0.248	4.04	1	0.044 *	0.607	0.373	0.988
Smoking status of brothers and sisters (Yes)	0.077	0.236	0.10	1	0.743	1.080	0.681	1.714
Number of smoking friends	0.744	0.207	12.89	1	<0.001 *	2.105	1.402	3.159
Number of friends becoming drunk	0.313	0.184	2.88	1	0.089	1.367	0.953	1.961
Number of marijuana-smoking friends	0.550	0.273	4.05	1	0.044 *	1.733	1.015	2.960
Number of days since individual skipped school	0.384	0.091	17.81	1	<0.001 *	1.468	1.228	1.754
Education situation at the end of last semester	0.177	0.145	1.47	1	0.224	1.193	0.897	1.586
Age of first cigarette	0.135	0.118	1.30	1	0.254	1.144	0.908	1.442
Attempts at stopping smoking	0.567	0.273	4.32	1	0.038 *	1.763	1.033	3.008
Number of days practicing binge-drinking	0.137	0.107	1.63	1	0.201	1.147	0.930	1.414
Marijuana consumption	−0.037	0.319	0.01	1	0.907	0.964	0.515	1.802
Feelings of sadness	0.264	0.274	0.93	1	0.334	1.303	0.762	2.227
Suicidal thoughts	0.781	0.354	4.87	1	0.027 *	2.184	1.092	4.370
Knowledge about smoking effects (Yes)	−0.165	0.282	0.34	1	0.557	0.847	0.488	1.472
Constants	−3.300	0.835	15.63	1	0.000	0.037		

* significant predictor.

## Data Availability

Data available on request.
